# Improving Properties of a Novel β-Galactosidase from *Lactobacillus plantarum* by Covalent Immobilization

**DOI:** 10.3390/molecules20057874

**Published:** 2015-04-30

**Authors:** Rocio Benavente, Benevides C. Pessela, Jose Antonio Curiel, Blanca de las Rivas, Rosario Muñoz, Jose Manuel Guisán, Jose M. Mancheño, Alejandra Cardelle-Cobas, Ana I. Ruiz-Matute, Nieves Corzo

**Affiliations:** 1 Departamento de Biotecnología y Microbiología de Alimentos, Instituto de Investigación en Ciencias de la Alimentación CIAL (CSIC-UAM), Campus de la Universidad Autónoma de Madrid, Nicolás Cabrera 9, 28049 Madrid, Spain; E-Mail: r.benavente@csic.es; 2 Grupo de Biotecnología Bacteriana, Instituto de Ciencia y Tecnología de Alimentos y Nutrición, (ICTAN-CSIC), Juan de la Cierva 3, 28006 Madrid, Spain; E-Mails: jcuriel@ifi.csic.es (J.A.C.); blanca.r@ifi.csic.es (B.D.L.R.); rmunoz@ictan.csic.es (R.M.); 3 Departamento de Biocatálisis, Instituto de Catálisis y Petroleoquímica (ICP-CSIC), Marie Curie 2, Cantoblanco, 28049 Madrid, Spain; E-Mail: jmguisan@icp.csic.es; 4 Grupo de Cristalografía y Biología Estructural, Instituto de Química Física Rocasolano (IQFR-CSIC), Serrano 119, 28006 Madrid, Spain; E-Mail: xjosemi@iqfr.csic.es; 5 CBQF—Centro de Biotecnologia e Química Fina, Escola Superior de Biotecnologia, Centro Regional do Porto da Universidade Católica Portuguesa, Rua Dr. António Bernardino Almeida, 4200-072 Porto, Portugal; E-Mail: acardelle@porto.ucp.pt; 6 Departamento de Bioactividad y Análisis de Alimentos, Instituto de Investigación en Ciencias de la Alimentación CIAL (CSIC-UAM), Campus de la Universidad Autónoma de Madrid, Nicolás Cabrera 9, 28049 Madrid, Spain; E-Mail: ana.ruiz@csic.es

**Keywords:** β-galactosidase, *Lactobacillus plantarum*, immobilization, glyoxyl-agarose, oligosaccharides synthesis, lactose, lactulose

## Abstract

A novel β-galactosidase from *Lactobacillus plantarum* (LPG) was over-expressed in *E. coli* and purified via a single chromatographic step by using lowly activated IMAC (immobilized metal for affinity chromatography) supports. The pure enzyme exhibited a high hydrolytic activity of 491 IU/mL towards *o*-nitrophenyl β-d-galactopyranoside. This value was conserved in the presence of different divalent cations and was quite resistant to the inhibition effects of different carbohydrates. The pure multimeric enzyme was stabilized by multipoint and multisubunit covalent attachment on glyoxyl-agarose. The glyoxyl-LPG immobilized preparation was over 20-fold more stable than the soluble enzyme or the one-point CNBr-LPG immobilized preparation at 50 °C. This β-galactosidase was successfully used in the hydrolysis of lactose and lactulose and formation of different oligosaccharides was detected. High production of galacto-oligosaccharides (35%) and oligosaccharides derived from lactulose (30%) was found and, for the first time, a new oligosaccharide derived from lactulose, tentatively identified as 3'-galactosyl lactulose, has been described.

## 1. Introduction

β-Galactosidases are enzymes which are very useful as catalysts that present several interesting applications in the food industry such as in the hydrolysis of lactose in milk for the production of new dairy products with no lactose in their composition. The total elimination of lactose in milk is required to solve the problem of lactose intolerance in a large percentage of the world population (approximately 70%) [1,2]. Also the prevention of lactose crystallization [3] or the increase of sweetness during the preparation of ice creams, condensed milk, etc, are interesting advantages of this reaction.

Other uses of β-galactosidases have been focused on oligosaccharide synthesis, such as prebiotic galacto-oligosaccharides (GOS) (e.g., from cheese whey lactose) [4,5,6]. Several β-galactosidases from different sources (*Kluyveromyces lactis*, *Bacillus circulans*, *Aspergillus niger*, *Eschericia coli*, *etc*…) are nowadays commercially available and some of them have been applied in the synthesis of glycosylated molecules [7].

However, in many cases, the use of β-galactosidases is limited by their low specific activity, low thermostability or high inhibition by reaction byproducts [8]. Therefore, the search and characterization of new and best β-galactosidases in generally recognized as safe (GRAS) microorganisms is an important topic. Among GRAS microorganisms, lactic acid bacteria are an adequate source of β-galactosidase enzymes. *Lactobacillus plantarum* is a lactic acid bacterium abundant in fermenting foods which also might colonize the human gastrointestinal tract. Recently, a tannase from this microorganism was successfully produced and applied in the hydrolysis of tannic acid to obtain gallic acid [9] for food preservatives [10]. β-Galactosidases from *L. plantarum* have been previously characterized [11,12,13], although a recently described glycosidase which exhibits β-galactosidase activity still remains uncharacterized [14].

A detailed characterization and optimization of β-galactosidases could give a better indication of their potential: (i) low inhibition by reaction products that could permit the full elimination of lactose in a short time; (ii) no dependency on cations to make them more versatile for the hydrolysis of different types of milk and whey; (iii) applicability under a broad pH range for the hydrolysis of lactose from acid whey; (iv) optimized transglycosylation processes; and (iv) enhanced thermal stability (e.g., by immobilization methods).

The purification of β-galactosidases is a key step in their application to milk modification to avoid side-reactions, e.g., by modification of other components of the milk by proteases [15]. Therefore the production of *L. plantarum* enzymes in *E. coli* combined with the purification of recombinant enzymes with a poly-His tag could provide access to large amounts of a specific pure enzyme. Herein, the improvement of the catalytic properties of a novel β-galactosidase from *Lactobacillus plantarum* (LPG) by multipoint covalent immobilization on aldehyde-supports has been described. Besides, a study of oligosaccharide formation from hydrolysis and transglycosylation of lactose and lactulose by LPG activity has also been carried out.

## 2. Results and Discussion

### 2.1. Purification of the Enzyme

**Figure 1:**
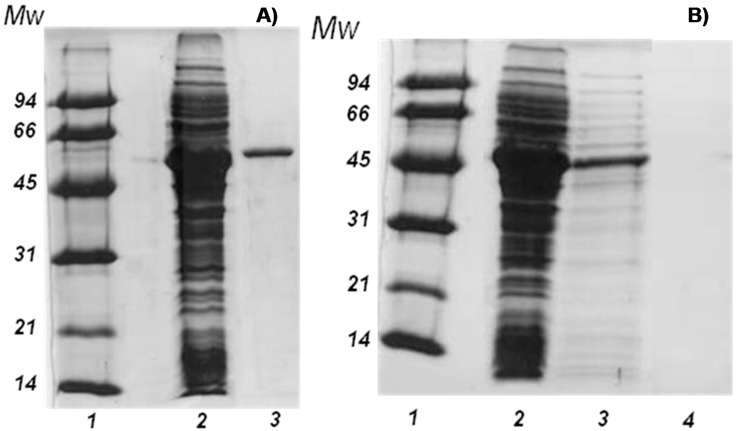
SDS-PAGE analysis of (**A**) LPG purification by adsorption on agarose-IDA-Ni support. Lane 1: Low molecular weight marker, Lane 2: Crude protein extract. Lane 3: Purified LPG and (**B**) different immobilized LPG preparations. Lane 1: Low molecular weight marker; Lane 2: Crude protein extract; Lane 3: Crude protein extract desorbed from CNBr support; Lane 4: Proteins desorbed from glyoxyl support.

Tailor-made agarose-IDA-Ni^2+^ supports were used to purify LPG. Purification yield was analyzed by SDS-PAGE according to the method of Laemmli [16]. The adsorbed protein extract showed a single band with a molecular weight corresponding to the β-galactosidase as shown in Figure 1.

The poly-His tagged β-galactosidase was fully adsorbed on the support for 10 min in the presence of 20 mM of imidazole to improve the selectivity of the adsorption process [17] (only 10% of undesired proteins from the total proteins contained in the crude extract were also adsorbed). After, LPG was eluted directly from the matrix using 100 mM of imidazole (Figure 1). The results of typical purification procedure are summarized in Table 1. The purification factor was 11.5 fold with a specific activity of 491 U/mg for *o*NPG. One single band at 52 kDa corresponding to the purified subunits of LPG from *Lactobacillus plantarum* was found on the SDS-PAGE gel (Figure 1A). As we have previously reported, this enzyme is an oligomeric species in solution with an average molecular weight of 390 kDa as determined by analytical gel-filtration chromatography [13]. This result agrees well with the crystallographic protein packing that indicates the existence of a hexameric assembly of 54.5 kDa subunits.

**Table 1:** Purification of β-galactosidase from *L. plantatum* (LPG).

Step	Activity (UI/mL)	Protein (mg/mL)	Recovered Activity (%)	Specific Activity (UI/mg)	Purification Factor
Crude extract	469.7	11	100	42.7	1
Ag-IDA-Ni^2+^	365.2	0.74	84	491.2	11.5

### 2.2. Biochemical Characterization of the β-Galactosidase from Lactobacillus plantarum (LPG)

Several biochemical properties of soluble and purified LPG were studied. The effect of the presence of different additives on the enzymatic activity was first evaluated (Table 2). Most tested cations did not affect the activity of soluble LPG and only the presence of Mn^2+^ caused a decrease in the activity. Therefore, it seems that the enzyme is stable in the presence of different additives. This is an advantage compared for example with the commercial β-galactosidase from *Kluyveromyces lactis* (Novozymes) which was quite sensitive to the presence of different additives, particularly with the presence of EDTA where a complete loss in activity was observed [18].

**Table 2:** Effect of different additives on the activity of β-galactosidase from *Lactobacillus plantarum* (LPG). Enzymes were incubated with 10 mM of each compound during 1 h at pH 7.

Additives	Relative Activity (%)
-	100
MnSO_4_	20
CaCl_2_	110
MgSO_4_	105
EDTA	99
NaH_2_PO_4_	99
KH_2_PO_4_	99

**Figure 2:**
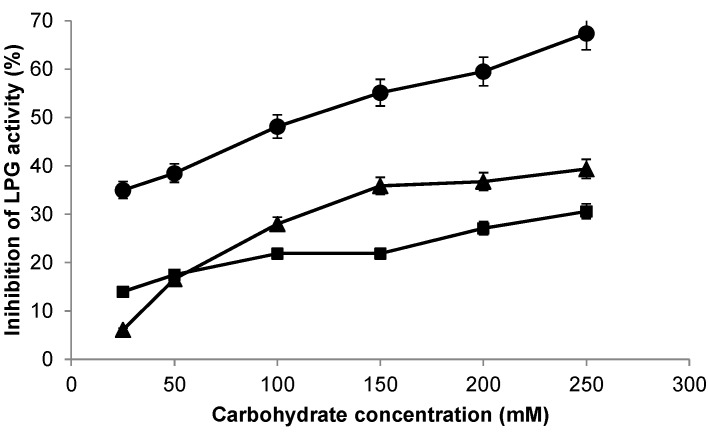
Inhibition of β-galactosidase from *Lactobacillus plantarum* (LPG) by different sugars. The reaction was carried out at pH 7, as described in the Experimental Section. Glucose (triangles), galactose (squares), lactose (circles).

In order to use these enzymes for lactose hydrolysis in milk, the effect of lactose concentration (from 25 to 250 mM) and resulting hydrolysis products such as glucose and galactose (from 25 to 250 mM) on the inhibition of free β-galactosidase activity was studied. As it can be appreciated in Figure 2, the enzyme was slightly inhibited by glucose and galactose at low concentration (5%–14% at 25 mM), while lactose inhibited 35% activity of the enzyme at this concentration. The inhibition profiles by these carbohydrates differed as their concentration increased (Figure 2). LPG exhibited similar inhibition with 50 mM or 250 mM of galactose (31% inhibited activity), whereas higher inhibition was achieved by using high concentrations of glucose, as a 40% of inhibition was found at 250 mM. As shown in Figure 2, lactose seems to be the best LPG inhibitor, as a 70% inhibition was achieved at 250 mM of substrate. These activity results obtained with LPG represent an advantage compared to the commercial β-galactosidase from *Kluyveromyces lactis* which was much more sensitive to these conditions [14,15,19].

### 2.3. Stabilization of LPG by Covalent Immobilization

The immobilization of the novel LPG on glyoxyl-agarose by multipoint covalent attachment was performed. The two supports gave 49.97 U/gram attachments, and in both cases more than 95% of the initial activity was immobilized. Once derivatives were obtained, we had positive proof that actually much of the enzyme was immobilized on both supports. To check the total enzyme activities expressed on the derivative, a small quantity of derivative (0.175 g) was dissolved in 2 mL of the respective buffer and the resulting enzyme activity was analyzed.

The final activity expressed was around 94%, so we assume that 100% of the initial activity would have been expressed in the derivative. After that, the stability of this immobilized biocatalyst was compared to a one-point covalent immobilized enzyme on CNBr-Sepharose support (an immobilized form similar to soluble enzyme).

**Figure 3:**
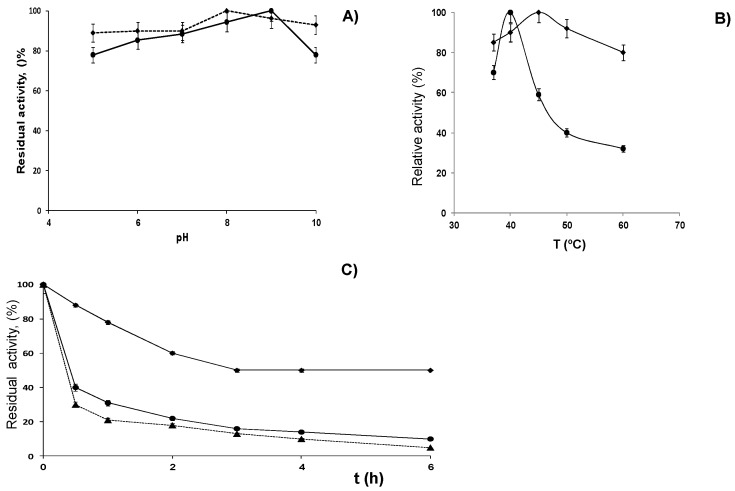
Influence of pH (**A**) and temperature (**B**) on residual activity of different preparations of β-galactosidase from *Lactobacillus plantarum* (LPG); Glyoxyl-LPG (rhombus), CNBr-LPG (one-point immobilized) (circles); and thermostability (**C**) at pH 7.0 and 50 °C of different preparations of LPG: Glyoxyl-LPG (rhombus), CNBr-LPG (one-point immobilized) (circles), soluble enzyme (triangles).

First, the stability of these immobilized preparations of LPG was checked in a range of pH or temperatures (Figure 3A,B, respectively). The glyoxyl-LPG preparation was very stable in the pH range from 5 to 9 with an optimum at pH 8. Similarly, the CNBr-LPG preparation or soluble enzyme was also quite stable up to pH 9, with just 20% loss of activity at pH 10 after incubation for 2 h. The highest activity of these biocatalysts was achieved at pH 9 (Figure 3A).

The optimal temperature of β-galactosidase activity of glyoxyl-LPG and CNBr-LPG is shown in Figure 3B. The optimal temperature of LPG after covalent immobilization on glyoxyl-agarose was 45 °C and 40 °C for CNBr-LPG prepared by one-point covalent immobilization. Furthermore, the glyoxyl-LPG derivative retained excellent activity up to 60 °C whereas the other preparations lost more than 60% of the activity at this temperature. Glyoxyl-LPG was stable in a wide range of temperatures.

Considering these results, the next experiments were carried out to study LPG stability by incubation for longer times at 50 °C and pH 7 (Figure 3C). The glyoxyl-LPG preparation maintained around 50% activity after 6 h, whereas one-point-CNBr-LPG and soluble LPG enzyme were practically fully inactivated (10%–15% remaining activity for both) at his time. Thus, the glyoxyl-LPG preparation was 6-fold more stable than the other analyzed preparations. Similar results were obtained when the thermal stability experiment was performed at pH 5 (data not shown).

All these results seem to indicate that this multipoint covalent attachment methodology allowed us to get a very stable LPG catalyst over a broad range of experimental conditions. This effect could be explained by a stabilization of the quaternary structure of the enzyme by this immobilization method and seems to corroborate that LPG enzyme presents several subunits as indicated previously [13] and similarly to other multimeric β-galactosidases [18,19,20].

In order to demonstrate this hypothesis, both LPG covalent immobilized preparations were boiled in the presence of SDS and mercaptoethanol and their supernatants were analyzed by SDS-PAGE (Figure 1B). After desorption of CNBr-LPG, a band corresponding to the β-galactosidase molecular weight was found (Figure 1B, lane 3) while no band was found on the SDS-PAGE pattern when the supernatant from desorption of glyoxyl-LPG was injected (Figure 1B, lane 4). Therefore, the results confirmed that some subunits are desorbed on the one-point covalent immobilization, so LPG seems to present several subunits (at least two) and the immobilization on glyoxyl-agarose permits the full immobilization and stabilization of all these subunits.

### 2.4. Synthesis of Oligosaccharides Derived from Lactose (GOS) and Lactulose (OsLu)

**Figure 4:**
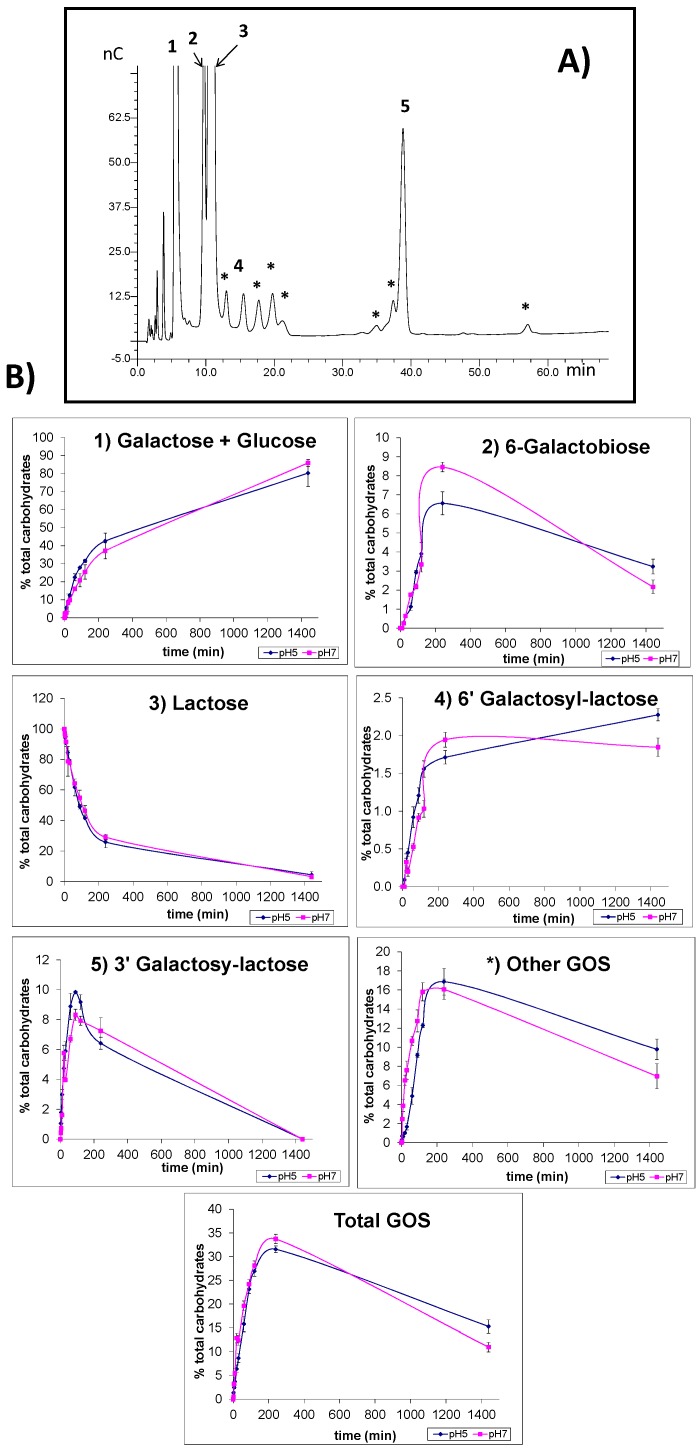
HPAEC-PAD profile of oligosaccharides from synthesis mixtures (**A**) and carbohydrate yields (%) (**B**) obtained during lactose hydrolysis by glyoxyl-immobilized β-galactosidase from *Lactobacillus plantarum* (LPG) at the two pH assayed (5 and 7). Compounds: (1) galactose + glucose; (2) 6-galactobiose; (3) lactose; (4) 6'-galactosyl-lactose; (5) 3'-galactosyl-lactose; (*) other oligosaccharides (GOS). Total GOS includes 6-galactobiose, 6'-galactosyl-lactose, 3'-galactosyl-lactose and other GOS. Squares: pH 7; rhombus: pH 5.

**Figure 5:**
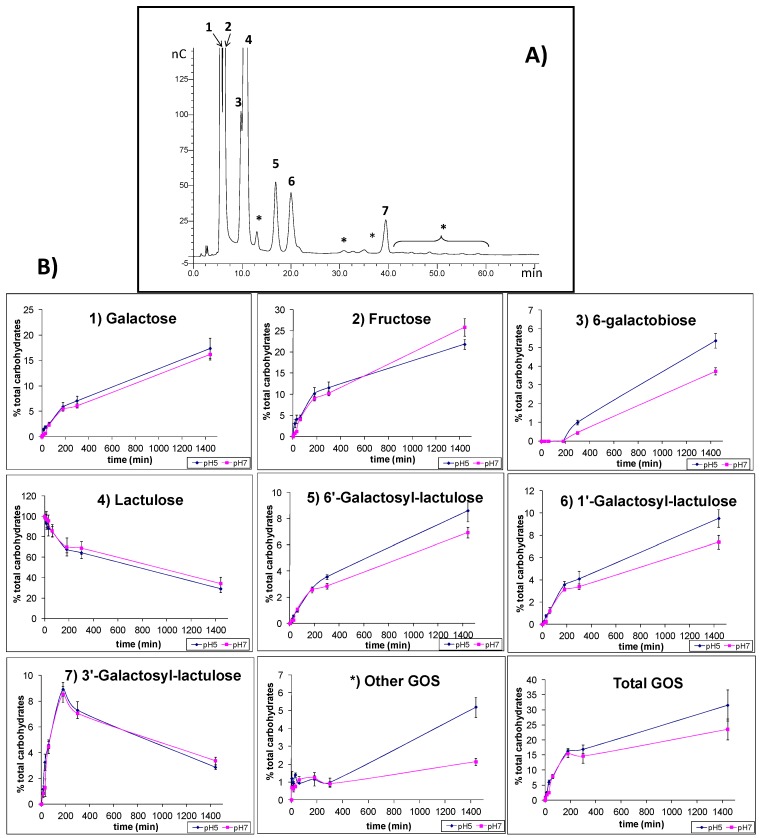
HPAEC-PAD profile of oligosaccharides from synthesis mixtures (**A**) and carbohydrate yields (%) (**B**) Obtained during lactulose hydrolysis by glyoxyl-immobilized β-galactosidase from *Lactobacillus platarum* (LPG) at the pHs assayed (5 and 7). Compounds: (1) galactose; (2) fructose; (3) 6-galactobiose; (4) lactulose; (5) 6'-galactosyl-lactulose; (6) 1-galactosyl-lactulose; (7) 3'-galactosyl-lactulose; (*) other oligosaccharides derived from lactulose (OsLu). Total OsLu include 6-galactobiose, 6'-galactosyl-lactulose, 1-galactosyl-lactulose, 3'-galactosyl-lactulose and other OsLu. Squares: pH 7; rhombus: pH 5.

The hydrolytic capacity of glyoxyl-immobilized LPG was evaluated using lactose and lactulose as substrates. Figure 4 and Figure 5 show the HPAEC-PAD carbohydrate profiles obtained from lactose and lactulose hydrolysis as well as their evolution during the hydrolysis process; the loss of lactose and lactulose; the release of galactose, glucose and fructose as well as the formation of the corresponding oligosaccharides.

The chromatographic profile resulting from the analysis of a reaction mixture of lactose (Figure 4A) shows the presence of galactose and glucose (peak 1); 6-galactobiose (peak 2); unreacted lactose (peak 3) and 6'-galactosyl lactose (peak 4), an unknown trisaccharide (peak 5), and unknown oligosaccharides (marked with an asterisk). Peak 5 was tentatively identified as 3'-galactosyl lactose by comparing its relative retention time (RRT), calculated against the retention time of lactose, with that found by Spelchtna *et al*. [21]. These authors studied the production of GOS from lactose using β-galactosidases from *Lactobacillus reuteri* and indicated that the tested strains, L103 and L461, have a high specificity for the formation of β (1→6) linkages, being β (1→3) linkages the second most important. In order to confirm the identification of 3'-galactosyl lactose, the synthesis mixture was analyzed by GC-MS and spectra of the TMSO derivatives of carbohydrates were recorded. The spectrum obtained for the peak previously assigned as 3'-galactosyl lactose (*m/z* values (abundances): 73 (62), 147 (38), 204 (100), 217 (49), 243 (10), 271 (8), 319 (12), 361 (61), 451 (4), 538 (12) was found to be compatible with the spectrum of other trisaccharides with 1→3 glycosidic bonds (nigerotriose and laminaritriose), confirming the proposed structure.

Figure 4B shows the evolution of carbohydrate and oligosaccharide formation throughout the reaction at the two pH values assayed. Lactose was hydrolyzed to monosaccharides (galactose + glucose) which levels increased with the reaction time. As it can be observed synthesis of trisaccharides predominated over other oligosaccharides. Among the GOS formed, the main trisaccharide was 3'-galactosyl-lactose, showing a maximum value of 10 g/100 g of total carbohydrates after 60 min of incubation at pH 5. The higher 6'-galactosyl lactose amounts were reached after 4 h of reaction, obtaining values of 2 g/100 g of total carbohydrates. Also, a high amount (16%) of unknown GOS was detected which contributed to the total GOS content achieving a maximum value of 34 and 32 g/100 g of total carbohydrates during 4 h of incubation at pH 7 and 5, respectively. At this time of course of reaction the hydrolysis of lactose was of 80%. The formation of GOS was similar in both pH values assayed.

The chromatographic profile of oligosaccharides formed during enzymatic hydrolysis of lactulose using LPG is shown in Figure 5. Peaks 1, 2, 3, and 4 were assigned to galactose, fructose, 6-galactobiose and lactulose, respectively. Peaks 5 and 6 corresponded to the two trisaccharides, 6'-galactosyl lactulose and 1-galactosyl lactulose, respectively. Besides, the presence of several unknown oligosaccharides which are marked with an asterisk was detected. Peak 7, which was the most abundant trisaccharide, was tentatively identified as 3'-galactosyl lactulose, since its isomer 3'-galactosyl lactose eluted at this retention time. The HPEAC-PAD profile of oligosaccharides produced by LPG was similar to that obtained by β-galactosidase from *Kluyveromyces lactis* [22,23,24] during lactulose hydrolysis however this new trisaccharide was not identified.

In order to confirm the identification, the sample was analyzed by GC-MS and, as occurred with 3'-galactosyl lactose, the spectra obtained for the peak previously assigned as 3'-galactosyl lactulose (*m/z* values (abundances): 73 (56), 147 (31), 204 (100), 217 (50), 243 (11), 271 (8), 319 (25), 361 (60), 451 (3), 538 (2)) was found to be compatible with the spectrum of carbohydrate standards with 1→3 glycosidic bonds.

The formation of oligosaccharide derivatives of lactulose (OsLu) as well as the remaining lactulose and released galactose and fructose during the time course of the reaction, at the two studied pH values, is shown in Figure 5B. The two trisaccharides 6'-galactosyl lactulose and 1-galactosyl lactulose were formed in similar amounts. The other trisaccharide, 3'-galactosyl-lactulose rapidly increased achieving its maximum value (9 g/100 g of total carbohydrates) during the 3 first hours of incubation. The highest yield of OsLu was produced after 24 h of reaction at pH 5 and pH 7, reaching values of 32 and 24 g/100 g of total carbohydrates, respectively. Similar behavior was observed at the two pH values studied.

The comparison of Figure 4 and Figure 5 shows that lactulose hydrolysis was slower than that of lactose. The maximum level of OsLu (32 g/100 g of total carbohydrates) was reached at 24 h of reaction while in the case of GOS the higher yield (34%) was attained after 4 h of reaction being the hydrolysis of lactose of 70%.

In this work a novel β-galactosidase from *L. plantarum* has been highly stabilized by immobilization on highly activated glyoxyl-agarose *via* a multipoint covalent attachment. This LPG immobilized preparation conserved activity over a broad range of pHs and temperatures, and it was 20-fold more stable than soluble enzyme or one-point covalent attached enzyme preparation in thermal inactivation tests at 50 °C. This biocatalyst was successfully used as catalyst in the hydrolysis and transglycosylation of lactose and lactulose producing high yields of prebiotic oligosaccharides. Among the obtained oligosaccharides, the presence of 3'-galactosyl lactulose which has been described for the first time as a new oligosaccharide derived from lactulose must be pointed out. Therefore this lactase represents a promising catalyst for applications in food chemistry and a valuable transglycosylase for the synthesis of prebiotic oligosaccharides.

## 3. Experimental Section

### 3.1. Chemicals

β-galactosidase from *Kluyveromyces lactis* (KLG) was purchased by Novozymes (Baggsvaerd, Denmark). The novel β-galactosidase from *Lactobacillus plantarum* (LPG) (protein contained an N-terminal six-histidine affinity tag) was produced from *E. coli* cells as previously described [13]. Cross-linked agarose 4BCL was purchased from GE Healthcare (Bio-sciences AB, Uppsala, Sweden). Agarose-IDA-Ni^2+^ supports were prepared as previously described [20]. Calcium chloride, ethylenediaminetetraacetic acid (EDTA), manganese sulphate, magnesium sulphate, *o*-nitrophenyl-β-d-galactopyranoside, (*o*-NPG), sodium borohydride, manganese sulphate, CaCl_2_, MgSO_4_ and EDTA, were from Sigma (St. Louis, MO, USA). Coomassie (Bradford) protein assay kit was purchased from Pierce (Waltham, MA, USA). CNBr-activated sepharose 4BCL support was from GE Healthcare. Glyoxyl-agarose supports were prepared as previously described [20]. Protein concentration was determined according to the procedure of Bradford [25] using bovine serum albumin as protein standard. Lactose was obtained from Scharlau (Barcelona, Spain). Fructose, galactose, glucose, lactulose, laminaritriose, nigerotriose and raffinose were purchased from Sigma-Aldrich Co (Steinheim, Germany). Galactose was acquired from Fluka (Steinheim, Germany). 6-*O-*β-Galactosyl-galactose (6-galactobiose) was from Carbosynth (Berkshire, UK). 6'-Galactosyl-lactose, 6'-galactosyl-lactulose, and 1-galactosyl-lactulose were standards prepared in our laboratory [22].

### 3.2. Enzymatic Activity Assays

The activities of the soluble β-galactosidase, supernatant and enzyme suspension were analyzed spectrophotometrically measuring the increment in absorbance at 405 nm produced by the release of *o*-nitrophenol (*o*NP) (∈ = 3.100 M^−1^ cm^−1^) in the hydrolysis of 13.3 mM *o*NPG in 50 mM sodium phosphate buffer in different conditions (aqueous phase or in the presence of different additives) at pH 7 and 25 °C. To initialize the reaction, enzyme solution or suspension (0.05–0.2 mL) was added to substrate solution (2 mL) under magnetic stirring. Enzymatic activity is given as one μmol of *o*-nitrophenol released per minute per mg of enzyme (IU) under the conditions described above.

### 3.3. Purification of β-Galactosidase from Lactobacillus plantarum (LPG)

Crude extract from *E. coli* containing LPG (10 mL) was diluted two times with 5 mM sodium phosphate buffer at pH 7.0 to a concentration of 11 mg/mL. Then, one gram of IDA-Ni^2+^-agarose support was added to the protein solution. Finally imidazole (25 mM final concentration, to prevent non-natural proteins from the extract from being adsorbed on the support) was also added. The mixture was gently stirred for 2 h at 25 °C. Periodically, the activity of suspensions and supernatants were measured by the *o-*NPG assay. Finally the mixture was filtered by vacuum and the solid was incubated on a solution of 50 mM sodium phosphate buffer with 100 mM imidazole to elute the adsorbed tagged enzyme, and tested by measure enzymatic activity using the method described above. Purification yield was analyzed by SDS-PAGE according to the method of Laemmli [16].

### 3.4. LPG Immobilization Process

A solution of pure enzyme (0.74 mg/mL) in 25 mM of the corresponding buffer as described in Section 3.3 was used in each case. The immobilization process was followed by the previously described enzymatic assay.

#### 3.4.1. Immobilization on CNBr-Activated Sepharose

To carry out the process of immobilization on CNBr, two procedures were used. To perform comparison tests between the derivatives in terms of temperature stability and/or pH, the CNBr derivative can be prepared with low loading running the reaction at 4 °C, so we avoid possible multipoint covalent interactions. Under these conditions, this derivative behaves like the free enzyme. The principal objective is not to use the crude extract, because with crude extract it is possible for different interactions take place, for example, protein-protein and this can promote possible stabilization of the derivatives promoting a false positive stability, so to prepare this derivative, the reaction time is quite short (10 min) and the reaction is run cold to get a one point derivative that completely simulates the properties of the free enzyme.

However, when we need a CNBr derivatized for direct use in a hydrolysis or synthesis reaction, a CNBr derivative with the maximum enzymatic loading has been prepared under standard conditions; (high capacity and covalent multipoint attachment) to promote greater stability against T and/or extreme pH values to obtain the maximum reaction yields.

The immobilization of LPG on CNBr-Sepharose was performed for 15 min at 4 °C to reduce the possibilities of a multipoint covalent attachment between the enzyme and the support. β-galactosidase solution (46.97 U, 10 mL), were added to one gram of support and the reaction was maintained for 1 h. Periodically, the activity of suspensions and supernatants was measured by the *o-*NPG assay. The enzyme-support immobilization was ended by incubating the support with 1M ethanolamine at pH 8 for 2 h. Finally, the immobilized preparation was washed several times with washing solution (0.1 M sodium bicarbonate, 0.5 M NaCl at pH 8.4) and afterwards with abundant water. The immobilization yield was >95%.

#### 3.4.2. Immobilization on Glyoxyl-Agarose

Crude enzyme solution (46.97 U, 10 mL), was added to 100 mM sodium bicarbonate buffer at pH 10.5 and the pH of the final solution was adjusted to pH 10.1. Then, one gram of glyoxyl-agarose (aldehyde activated support) was added and the reaction was maintained for 24 h. Periodically, activity of suspensions and supernatants were measured by using the *o*NPG assay. When the immobilization was finished, NaBH_4_ (10 mg) was added for 30 min and then the suspension was filtered and washed abundantly with distilled water (200 mL × 5). The immobilization yield was >95%.

### 3.5. Effect of pH and Temperature on Enzyme Activity

The pH dependency of enzyme activity was determined at 25 °C in three different buffers—sodium citrate, phosphate buffer (50 mM) from pH 5.0 to 6.0, sodium potassium phosphate buffer (50 mM) from pH 7.0 to 8.0 and sodium bicarbonate buffer (50 mM) from pH 9.0 to 10.0—using the assay described above. The effect of temperature on enzyme activity was determined at temperatures ranging from 30 to 70 °C. Aliquots were withdrawn at 0.5 to one hour intervals.

The different LPG enzyme preparations (soluble or immobilized enzyme) were used. Samples were periodically withdrawn using a pipette with a cut-tip and under vigorous stirring in order to have a homogeneous biocatalyst suspension and the activity was assayed as described before. The experiments were carried out by triplicate and the standard error was under 5%. In each case, the initial activity was considered to be the 100% value.

### 3.6. Effect of Sugars Concentrations on LPG Kinetic Parameters

The potentially inhibiting effects of lactose, glucose and galactose on LPG activity were investigated separately. For the β-galactosidase inhibition analysis both soluble and immobilized preparations of the enzyme were evaluated at different concentrations (from 10 mM to 1M) of the reaction products.

### 3.7. Synthesis of Oligosaccharides Derived from Lactose (GOS) and Lactulose (OsLu)

Synthesis of oligosaccharides derived from lactose (GOS) and lactulose (OsLu) were carried out using 300 g/L of lactose or 450 g/L of lactulose solutions. Two different buffers with two different pH, (sodium acetate buffer pH 5 and sodium phosphate buffer pH 7) were assayed. One gram of each derivative (containing 5 U/mL of LPG-enzyme) was incubated at 45 °C up to 24 h. Lactose and lactulose solutions were heated before the enzyme extract was added and were maintained at the required temperature throughout the experiment. During the reaction, the pH value was maintained constant using a pH-stat Mettler Toledo DL50 graph. Reactions were performed in individual Eppendorf tubes and incubated in an orbital shaker at 400 rpm. Samples of 200 μL were withdrawn from the reaction mixtures at different times and immediately immersed in boiling water for 5 min to inactivate the enzyme. After appropriate dilution, 20 μL were injected into the chromatograph. All experiments were performed in duplicate.

### 3.8. Analytical Techniques

#### 3.8.1. SDS-PAGE Analysis of the Free and Immobilized Enzyme

Proteins were analyzed by SDS-PAGE according to Laemmli [16], using a precast 12% gradient gel (model protean 16; Bio-Rad Laboratories, Richmond, CA, USA). Gels were stained with Coomassie brilliant blue R-250. To analyze the amount of proteins adsorbed on IMAC supports, a sample of the support was first boiled in the presence of 1% SDS (w/v) and 2% 2-mercaptoethanol (v/v) to desorb the proteins. Low molecular weight markers from GE Healthcare were used (14–94 kDa).

#### 3.8.2. Chromatographic Determination of Oligosaccharides Derived from Lactose (GOS) and Lactulose (OsLu)

##### High-Performance Anion Exchange Chromatography with Pulsed Amperometric Detection (HPAEC-PAD) Analysis

GOS and OsLu were determined by high-performance anion exchange chromatography with pulsed amperometric detection (HPAEC-PAD) on a ICS2500 Dionex system (Dionex Corp., Sunnyvale, CA, USA) consisting of a GP50 gradient pump and ED50 electrochemical detector with a gold working electrode and Ag/AgCl reference electrode. Separations were performed following the method described by Splechtna *et al.* [21]. Elution was at room temperature on a CarboPac PA-1 column (250 × 4 mm) connected to a CarboPac PA-1 (50 × 4 mm) guard column. Eluent A (100 mM NaOH), eluent B (100 mM NaOH and 50 mM NaOAc) and eluent C (100 mM NaOH and 1 M NaOAc) were mixed to form the following gradient: 100% A from 0 to 20 min and 0% to 100% C from 20 to 70 min. After each run, the column was washed for 10 min with 100% B and re-equilibrated for 15 min with the starting conditions of the employed gradient. Separations were performed at a flow rate of 1 mL/min. Detection time and voltage parameters were set as follows: E_1_ = 0.1V (t_1_ = 400ms), E_2_ = 2.0V (t_2_ = 10ms), E_3_ = 0.6V, E_4_ = −0.1V (t_4_ = 60ms); t_t_ = 500 ms. Samples and standard solutions were filtered through a nylon Millipore FH membrane (0.22 μm) (Bedford, MA, USA) before injection. Data adquisition and processing were performed with Chromeleon 6.7 software (Dionex Corp.). Quantification of carbohydrates was performed by external calibration using standard solutions of galactose, fructose, lactose, lactulose and raffinose. The regression coefficients of the curves for each standard were always greater than 0.99. The amount of lactose or lactulose remaining and the yield of GOS and OsLu were expressed as g/100 g of the total carbohydrate content in the reaction mixtures.

##### Gas Chromatographic-Mass Spectrometric (GC-MS) Analysis

GC-MS analysis of carbohydrates was carried out using a Agilent Technologies 7890A gas chromatograph coupled to a 5975C MSD quadrupole mass detector operating in electron impact (EI) mode at 70 eV (both from Agilent, Palo Alto, CA, USA). Analyses were carried out in split mode (1:40) on a bonded fused silica capillary column SPB-17, crosslinked phase (50% diphenyl/50% dimethylsiloxane; 30 m × 0.32 mm i.d., 0.5 μm film thickness) (Supelco, Bellefonte, PA, USA), heated at 180 °C to 290 °C at a heating rate of 3 °C min^−1^ and held for 35 min. Injector temperature was 280 °C and the transfer line was at 250 °C. Helium at ~1 mL min^−1^ was used as carrier gas. Acquisition was done using an Agilent ChemStation MSD (Agilent). Previous to injection, carbohydrates were converted into their trimethyl silylated oximes (TMSO) derivatives following the method described by Brobst and Lott [26].

## 4. Conclusions

In this work, we report the ability to produced, purified, immobilized and stabilized the new beta-galactosidase and characterized biochemically to use its derivative in the synthesis of galactooligossacharides from lactose and lactulose acting as subtract for this kind of reaction.
